# Inappropriate Birth Weight for Gestational Age Among Newborns Born at Dessie Referral Hospital: A Retrospective Cohort Study

**DOI:** 10.1155/ijpe/3491139

**Published:** 2024-12-20

**Authors:** Setegn Mihret, Kalkidan Wondwossen, Rodas Merid, Ketema Bizuwork Gebremedhin

**Affiliations:** ^1^Department of Pediatrics and Child Health Nursing, College of Medicine and Health Science, Wollo University, Dessie, Ethiopia; ^2^School of Nursing and Midwifery, College of Health Sciences, Addis Ababa University, Addis Ababa, Ethiopia; ^3^Department of Emergency and Critical Care, St. Paulos Millennium Medical College, Addis Ababa, Ethiopia

**Keywords:** Ethiopia, inappropriate birth weight for gestational age (IBWGA), large for gestational age (LGA), small for gestational age (SGA)

## Abstract

Inappropriate birth weight for gestational age (IBWGA) is linked with obstetric complications like birth asphyxia, hypothermia, and postpartum hemorrhage. This study was aimed at determining the prevalence of IBWGA with factors associated with newborns born at Dessie Referral Hospital, northeast of Ethiopia. We used a retrospective cohort study design and systematic random sampling method to select charts of women giving birth at the hospital from January 2013 to December 2017. Binary logistic regression analysis was used to check the association of selected variables with the outcome variable IBWGA. The prevalence of IBWGA was found to be 145 (34.36%), with 52 (12.32%) and 93 (22.04%) for large for gestational age (LGA) and small for gestational age (SGA), respectively. A higher prevalence of IBWGA was found among women who use substances during pregnancy, such as chewing chat (43, 49.4%), smoking (14, 53.8%), and those with a history of giving birth to an infant with IBWGA (31, 50.0%). Furthermore, maternal age less than 35 years old (*p* < 0.05), antenatal care initiation at or before the second trimester (*p* < 0.05), gestational age less than 37 weeks (*p* < 0.05), and chewing chat during pregnancy (*p* < 0.05) were found to be statistically significantly associated with IBWGA. The high prevalence of IBWGA revealed by this study suggests a need for interventions focusing on its predicting factors: maternal age, prenatal care, gestational age, and substance use during pregnancy.

## 1. Introduction

Inappropriate birth weight for gestational age (IBWGA) is defined as birth weight below the 10th percentile (small for gestational age (SGA)) or above the 90th percentile (large for gestational age (LGA)), referring distribution of weights to gestational ages [1, 2]. IBWGA is a major predictor of neonatal mortality and morbidity like failure to thrive, asphyxia, neurodevelopment delay, congenital heart diseases, traumatic deliveries, stillbirth, and low Apgar score [3–7]. Among the more than 19% of SGA babies in low- and middle-income countries—predicted to be over 23 million—roughly 22% were estimated to be dying [8]. The prevalence of SGA in sub-Saharan Africa was reported to be 16.5%, while LGA in certain Western and East African countries ranges from 16% to 34% [3, 9–12]. In sub-Saharan countries, SGA and LGA accompany neonatal mortality are growing simultaneously and are unacceptably outpaced in some East African countries with the highest neonatal mortality [10, 13, 14]. Even though progressive improvement was observed in reducing IBWGA and its adverse outcomes through the implementation of routine perinatal care services, the problem still exists as a healthcare burden across the globe, which is intense in developing nations. Hence, it is important to regularly monitor the multidimensional progress of the problem in order to prevent the magnitude of IBWGA and its adverse outcomes for the local and national communities. Therefore, this study was aimed at determining the prevalence and factors associated with IBWGA among newborns born at Dessie Referral Hospital, Northeast Ethiopia.

## 2. Conceptual Framework

**Figure 1:**
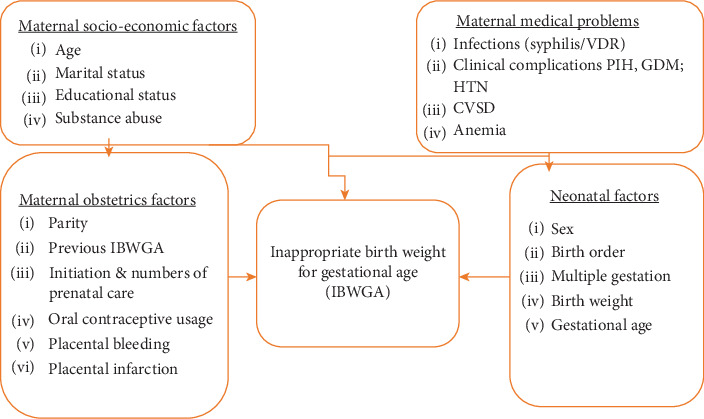
Conceptual framework shows factors affecting inappropriate birth weight (IBWGA). Abbreviations: VDR, Venereal Disease Research; PIH, pregnancy-induced hypertension; GDM, gestational diabetes mellitus; HTN, hypertension; CVS, cardiovascular disorder.

This study explored factors affecting IBWGA based on the health belief model which states people's beliefs affect the practice that helps them to prevent disease [15]. Thus, this conceptual framework states the effect of maternal sociodemographic characteristics and medical, obstetric, and neonatal factors (Figure 1).

## 3. Methods and Materials

### 3.1. Study Area, Study Design, Study Period, Study Participants, and Sampling Technique

This study was conducted at Dessie Referral Hospital, south Wollo zone of Amhara Regional State, northeast Ethiopia, from February 10 to March 10, 2018. Dessie City is found 401 km distant from the capital city of Ethiopia, Addis Ababa, and 484 km distant from Bahr Dar, the capital city of Amhara Regional State [16]. The city is the second most populated city and the corridor site of many entries in the Amhara Region [16], which has 26 kebeles (the lowest administrative level in Ethiopia). According to the 2021 population projection of the Ethiopian statistical agency, the city has about 257,126 population in the city [17].

Dessie Referral Hospital is the only referral hospital found in the south Wollo zone [18]. The hospital serves an average of 5107 births per year by the well-equipped neonatal intensive care unit (NICU). This study used a retrospective cohort study design to recruit the study participant's charts through a systematic random sampling technique by excluding charts with incomplete records of gestational age and weights.

### 3.2. Sample Size Determination

The sample size was determined by a single population proportion formula, considering a 95% confidence interval (CI), 5% marginal error, and 50% as the previous proportion of IBWGA. Therefore, the final sample size calculated was 422, including a 10% nonresponse rate.

### 3.3. Data Collection Methods and Quality Control

The structured and pretested checklist used to collect the data for the study was adapted from a study conducted elsewhere [19]. The checklist was validated by five experts. After we addressed the suggestions and recommendations of the experts, each expert's evaluation of questions was calculated for the content validity index and rated at 0.90. The data collectors and supervisors were trained in the way data could be collected. The supervisor of the study monitored the data collection process and managed the encountered difficulties on the spot.

### 3.4. Study Variables and Measurement

#### 3.4.1. Outcome Variable

IBWGA: Newborns' birth weight for gestational age is below the 10th percentile and above the 90th percentile recorded on the maternal chart which fulfills the cross-checking using hospitals' birth weight for gestational age reference graph and described by descriptive statistics.

#### 3.4.2. Independent Variables

Demographic characteristics of the newborn: these are gestational age, sex, weight, and gestations of the newborns; maternal sociodemographic characteristics: these are age, marital status, educational status, ethnicity, and substance abuse of women who participated in the study; maternal obstetric–related factors: these are factors like parity, previous delivery of IBWGA, gestational weight gain, threatened preterm labor, entry time into prenatal care, and oral contraceptive usage; and maternal medical disorders related factors: these include the anemic status of the women, history of hypertension, cardiovascular system disorder, diabetes mellitus, gestational diabetes mellitus, and syphilis, described by descriptive statistics.

#### 3.4.3. Ethical Approval

We obtained a letter of ethical clearance and a support letter from the institutional review board of the College of Health Sciences, Addis Ababa University. The medical record staff and data collectors accomplished, searched, and obtained the predetermined number of charts using a systematic random sampling method.

### 3.5. Methods Data of Analysis

The collected data was entered into EpiData Version 3.1 and analyzed by SPSS Version 20. In order to control some confounders, multivariate logistic regression analysis was used to investigate the association of selected variables with the dependent variable, IBWGA, with a level of significance of *p* < 0.05.

## 4. Results

### 4.1. Maternal Sociodemographic Characteristics

A total of 422 women charts were reviewed for this secondary data analysis study. The mean age of the women was 26.85 ± 6.002. The majority, 355 (84.1%), 234 (55.5%), 355 (84.1%), and 270 (64.0%), of the women were younger in age, were recruited from urban residents, married, and attended at least elementary educational level, respectively. Significant proportions, 26 (6.2%), 79 (18.7%), and 87 (20.6%), of the women were cigarette smokers, alcohol consumers, and chews chat, respectively (Table 1). Further, a significant proportion, 41 (9.7%), 44 (10.4%), and 62 (14.7%), of the women had a history of using combined oral contraceptives, experienced miscarriage, and had a history of being born a newborn with IBWGA, respectively (Table 1). The majority, 346 (82.0%) and 335 (79.4%), of the women started to attend antenatal care service centers before or during their second trimester of pregnancy and were primi/nulli para in their obstetric history, respectively (Table 1). Furthermore, significant women had a history of comorbidity with chronic diseases like HIV/AIDS (26, 6.6%), cardiovascular disease (2, 0.5%), hepatitis B infection (24, 5.7%), anemia (78, 19.1%), hypertension (44, 10.4%), diabetes mellitus (58, 15.4%), and syphilis (18, 4.4%). Regarding the demographic history of the newborns, significant proportions were born preterm (41, 9.7%), with birth defects (23, 5.5%), twin (32, 7.6%), and died at their postpartum period (19, 4.5%). Further, the majority (233, 55.2) of the newborns born were male in their sex (Table 1).

### 4.2. IBWGA and Associated Factors

**Figure 2:**
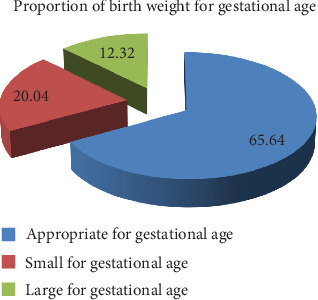
Proportion of birth weight for gestational age (*N* = 422).

The overall prevalence of IBWGA was 145 (34.36%), with LGA 52 (12.32%) and SGA 93 (22.04%) (Figure 2). The higher prevalence of IBWGA was among older women (≥ 35 years old) (34, 50.7%), rural residents (71, 37.8%), married (126, 35.5%), and illiterate (60, 39.5%) women who participated in the current study. Further, significant proportions of IBWGA were born from women who had a history of chewing chat (43, 49.4%), alcohol drinking (29, 36.7%), cigarette smoking (14, 53.8%), and using the combined oral contraceptive method (19, 46.3%) (Table 1).

Furthermore, significant proportions of newborns with IBWGA were born from women with a history of previous IBWGA (31, 50.0%) and a history of miscarriage (19, 43.2%). Moreover, this study found equivalent proportions of twin and singleton newborns were born being IBWGA. However, the higher proportions of IBWGA were from newborns born preterm (16, 39.0%) and males (67, 35.4%) in their sex (Table 1).

Noteworthy proportions, 35 (40.2%) and 42 (55.3%), of the newborns born IBWGA were from women who attended antenatal care centers over two times and were multipara in their obstetric history, respectively (Table 1).

All the women presented with histories of chronic diseases like diabetes mellitus, hypertension, cardiovascular disease, anemia, hepatitis B infection, and/or had a positive test history for syphilis and HIV/AIDS were given births of newborns with IBWGA. Furthermore, the birth weight of all the newborns who died and were born with some kind of birth defect was inappropriate.

Regarding the association of variables with the occurrence level of IBWGA, we used the odds ratio of the binary regression model with a significance level of *p* < 0.05. Therefore, this study revealed the age of the women being < 35 years old (odds ratio (OR) = 1.62 [95% CI: 1.02, 2.58], *p* < 0.05), having a history of starting to attend an antenatal care center below or at the second trimester (OR = 1.86 [95% CI: 1.20, 2.87], *p* < 0.05), and being born before 37 weeks of gestational age (OR = 9.94 [95% CI: 5.71, 17.29], *p* < 0.05) were found to be more likely to be linked with the occurrence of IBWGA, while having a history of chewing chat during pregnancy (OR = 0.62 [95% CI: 0.40, 0.94], *p* < 0.05) was found to be less likely to be linked with the occurrence of IBWGA (Table 1).

## 5. Discussion

This study revealed a significant (145, 34.36%) proportion of newborns were born with IBWGA, where the proportion of LGA was 52 (12.32%) and SGA was 93 (22.04%) at Dessie Referral Hospital from January 2013 to December 2017. The higher proportion of IBWGA revealed by the current study could be due to over/undernutrition, psychological stress, maternal height, weight, parity, and comorbidity of the women encountered during pregnancy [20, 21]. This signposts the higher concern that there could be higher later-age complications like diabetes, obesity, and neurological and cardiovascular disorders; likewise, it increases the chance of death of newborns and/or children under 5 years old [22, 23].

The higher the findings of the IBWGA revealed by the current study, is in fact lower than the study from northern California, which revealed 25.1% African American women, 17.3% Hispanic women, 16.4% white women, 15.3% Filipina women, and 13.9% Asian women were born newborns of LGA [24]. Furthermore, lower than studies from low- and middle-income countries which revealed 19.3%–41.5% of newborns were born SGA [1, 2, 25]. However, higher than a study from Tanzania, which revealed 15.8% of the newborns, were born SGA [13], and a study from Thailand which revealed about 2.6% and 10.5% of the newborns were born SGA and LGA, respectively [26]. Regarding the factors associated with IBWGA, this study found the younger the women were found to be more likely to give birth to newborns with IBWGA; this implies the older the women, the more likely they are to give birth to newborns with IBWGA. This finding is in line with a study from Qatar [27], however, contradicts study findings from the United States and the Netherlands and analysis of data at the global level [28–30].

This study revealed maternal conditions like women who had a history of chewing chat were found less likely to give birth to newborns with IBWGA. This finding contradicts study findings from Italy, the United States, and Pakistan [31–34]. However, contrary to a study from Israel [35], which revealed smoking cigarettes was positively associated with the occurrence of IBWGA. The difference could be due to the difference in sociodemographic characteristics of the study participants, even though substance use during pregnancy significantly affects the growth of the fetus and even leads to death [36]. Further, this study revealed being born preterm and IBWGA have strong links. This could be due to under/overnutrition of the mother that she experiences in her entire life and/or during pregnancy, which is a leading biological factor for IBWGA_SGA and face-flattering growth rate [12], which can be addressed/catch up during prenatal care, which is also an important determinant factor for IBWGA.

Further, this study revealed that all women with a history of chronic diseases like anemia, cardiovascular disease, diabetes mellitus, hypertension, and/or infected with sexually transmitted diseases HIV/AIDS, and syphilis were found to be given birth to newborns with IBWGA. The link of the anemia with IBWGA could be due to poor dietary condition of the women and/or poor absorption rate, which is important for the development/growth of the fetus [37], while the link for diabetes with IBWGA, with the most probable birth outcome LGA [38], could be due to the increase in placental transport of nutrients like glucose, amino acids, and fatty acids stimulating the fetus to produce endogenous insulin and insulin-like growth factor 1 that helps to utilize the nutrients and results in a large size of the fetus for gestational age [39].

Therefore, bearing in mind the extent of IBWGA including both the LGA and SGA revealed by the current study, the following interventions were suggested. Firstly, the government and stakeholders should increase the means of delivering appropriate information like underlying maternal health conditions, anemia, diabetes mellitus, and experience of harmful behaviors that help the community to prevent IBWGA. Secondly, the healthcare providers working in the prenatal department should update themselves with recent information about pregnancy and pregnancy-related care to be given to the women and should make aware the women in need of prenatal care about IBWGA for gestational and its benefits. Thirdly, the healthcare system should encourage screening of IBWGA at each antenatal care visit. The limitation of the study is the study design we used; a retrospective study leads to missing information due to the focus/unavailability difference of investigations and examinations from institution to institution. Therefore, it is important that future studies be studied prospectively with primary data.

## 6. Conclusion

This study found a higher proportion of newborns were born with IBWGA, with predicting factors being young in maternal age, being born preterm, being started to attend antenatal care before or at the second trimester, and chewing chat during pregnancy. Therefore, there should be inimitable interventional means that lessen the high prevalence of IBWGA and related complications.

## Figures and Tables

**Table 1:** Maternal and newborn demographic characteristics and their association with the extent of IBWGA (*N* = 422).

**Variables**	**Tested for IBWGA** **422**	**IBWGA** **145 (34.36)**	**OR (95% CI)**
*Maternal and newborn demographic characteristics*			
Age			
<35 year	355	111 (31.3)	1.62 (1.02, 2.58)⁣^∗^
≥35 year	67	34 (50.7)	
Residence			
Rural	188	71 (37.8)	0.84 (0.74, 1.22)
Urban	234	74 (31.6)	
Marital status			
Married	355	126 (35.5)	0.80 (0.46, 1.38)
Unmarried	67	19 (28.4)	
Educational status			
Illiterate	152	60 (39.5)	0.80 (0.54, 1.17)
Elementary and above	270	85 (31.5)	
History of chat chewing during pregnancy			
Yes	87	43 (49.4)	0.62 (0.40, 0.94)⁣^∗^
History of alcohol consumption during pregnancy			
Yes	79	29 (36.7)	0.92 (0.57, 1.48)
History of smoking history during pregnancy			
Yes	26	14 (53.8)	0.61 (0.31, 1.21)
History of oral COC usage			
Yes	41	19 (46.3)	0.71 (0.40, 1.27)
Previous history of giving birth to IBWGA			
Yes	62	31 (50.0)	0.63 (0.39, 1.02)
History of parity			
Nulli/primi para	335	110 (32.8)	1.23 (0.78, 1.92)
Multipara	87	35 (40.2)	
Started ANC attendance			
≤ Second TM	346	103 (29.8)	1.86 (1.20, 2.87)⁣^∗^
≥ Third TM	76	42 (55.3)	
Previous history of miscarriage			
Yes	44	19 (43.2)	0.77 (0.43, 1.37)
Number of newborns at current pregnancy			
Single	390	135 (34.6)	0.90 (0.43, 1.89)
Multiple	32	10 (31.3)	
Gestational age			
< 37 weeks	41	16 (39.0)	9.94 (5.71, 17.29)⁣^∗^
≥ 37 weeks	381	129 (33.9)	
Newborns' sex			
Male	233	78 (33.5)	1.06 (0.73, 1.55)
Female	189	67 (35.4)	

Abbreviations: COC, combined oral contraceptive; IBWGA, inappropriate birth weight for gestational age; TM, trimester.

⁣^∗^Statistically significant at *p* < 0.05.

## Data Availability

The data will be available from the corresponding author upon reasonable request.
